# Correcting an overactive exercise pressor reflex: A new role for purinergic signalling?

**DOI:** 10.1113/EP091733

**Published:** 2024-02-15

**Authors:** Fiona D. McBryde

**Affiliations:** ^1^ School of Medical Sciences University of Auckland Auckland New Zealand

**Keywords:** cardiovascular disease, exercise pressor reflex, purinergic signalling

1

The exercise pressor reflex is a fundamental physiological mechanism that evokes increases in blood pressure, heart rate and total peripheral resistance by adjusting autonomic tone to the heart and blood vessels. An exaggerated or overactive exercise pressor reflex is a common feature of peripheral artery disease (PAD)—patients with this condition show heightened reflex sensitivity, exaggerated blood pressure responses and reduced exercise tolerance, associated with impaired blood flow regulation and ischaemia in active skeletal muscle. Importantly, an exaggerated blood pressure (BP) response to exercise has been identified as a key marker for increased cardiovascular risk.

In this issue of *Experimental Physiology*, Qin et al. (2023) show an important role of purinergic signalling in mediating the exaggerated exercise pressor responses seen following ischaemia–reperfusion, using a hindlimb ischaemia–reperfusion challenge in rats as a preclinical model of PAD. The importance of purinergic signalling was demonstrated utilizing an array of molecular, electrophysiological and integrative in vivo techniques. First, western blot demonstrated that ischaemia–reperfusion leads to an upregulation of P2X_3_ expression in dorsal root ganglia—while previous work has shown a similar upregulation with prolonged hindlimb ischaemia, the current study confirms that P2X_3_ receptor levels remain elevated even after 18 h of recovery after an occlusion period, a scenario that mimics the clinical one of intermittent claudication, where the severity of ischaemia fluctuates with exertion. An important finding is a series of in vitro and in vivo experiments that demonstrate the functional consequences of this increase in P2X_3_ receptor expression with an amplification of P2X_3_ currents in dorsal root ganglia neurons and a P2X_3_‐mediated increase in BP during static muscle contraction. Importantly, this study goes on to use pharmacological and genetic blockade (siRNA) of P2X_3_ signalling pathways to identify a causal role of purinergic signalling in mediating the exaggerated EPR after hind limb ischaemia–reperfusion.

The putative role of purinergic signalling pathways in cardiovascular disease has received considerable interest in recent times. In particular, P2X_3_ receptors contained within the carotid body have been identified as potential therapeutic targets for hypertension (e.g., Pijacka et al., 2016) and heart failure (e.g., Lataro et al., 2023). While the current study focuses on the more localized purinergic upregulation in PAD, an abnormal exercise pressor reflex and subsequent sympathetic overactivation is a common feature of many cardiovascular conditions, including hypertension, diabetes and heart failure (Grotle et al., 2020). A key ambition is to identify whether treatment to ameliorate exaggerated exercise pressor responses could alleviate the associated cardiovascular risk burden. The current study by Qin et al. (2023) in combination with previous work in 2011 identifies the P2X_3_ receptor as a promising target, especially given the recent European Medicines Agency approval of the P2X_3_ antagonist gefapixant (Lyfnua™) for human use.

## AUTHOR CONTRIBUTIONS

Sole author.

## CONFLICT OF INTEREST

The author declares no conflicts of interest.

## FUNDING INFORMATION

No funding was received for this work.
